# Human Adipose-Derived Stem Cells in Madelung’s Disease: Morphological and Functional Characterization

**DOI:** 10.3390/cells10010044

**Published:** 2020-12-30

**Authors:** Federica Caponnetto, Ivana Manini, Michela Bulfoni, Nicola Zingaretti, Giovanni Miotti, Carla Di Loreto, Daniela Cesselli, Laura Mariuzzi, Pier Camillo Parodi

**Affiliations:** 1 Department of Medicine, University of Udine, 33100 Udine, Italy; michela.bulfoni@uniud.it (M.B.); carla.diloreto@uniud.it (C.D.L.); daniela.cesselli@uniud.it (D.C.); laura.mariuzzi@uniud.it (L.M.); piercamillo.parodi@uniud.it (P.C.P.); 2 Institute of Pathology, University Hospital of Udine, 33100 Udine, Italy; ivana.manini@tiscali.it; 3 Department of Medical Area (DAME), Clinic of Plastic and Reconstructive Surgery, Academic Hospital of Udine, University of Udine, 33100 Udine, Italy; zingarettin@gmail.com (N.Z.); gio.miotti@gmail.com (G.M.)

**Keywords:** Madelung’s disease, lipomatosis, Launois–Bensaude syndrome, human adipose-derived stem cells, mitochondrial mutations

## Abstract

Madelung Disease (MD) is a syndrome characterized by the accumulation of aberrant symmetric adipose tissue deposits. The etiology of this disease is yet to be elucidated, even though the presence of comorbidities, either genetic or environmental, has been reported. For this reason, establishing an in vitro model for MD is considered crucial to get insights into its physiopathology. We previously established a protocol for isolation and culture of stem cells from diseased tissues. Therefore, we isolated human adipose-derived stem cells (ASC) from MD patients and compared these cells with those isolated from healthy subjects in terms of surface phenotype, growth kinetic, adipogenic differentiation potential, and molecular alterations. Moreover, we evaluated the ability of the MD-ASC secretome to affect healthy ASC. The results reported a difference in the growth kinetic and surface markers of MD-ASC compared to healthy ASC but not in adipogenic differentiation. The most commonly described mitochondrial mutations were not observed. Still, MD-ASC secretome was able to shift the healthy ASC phenotype to an MD phenotype. This work provides evidence of the possibility of exploiting a patient-based in vitro model for better understanding MD pathophysiology, possibly favoring the development of novel target therapies.

## 1. Introduction

Madelung disease (MD), also known as multiple symmetric lipomatosis or Launois–Bensaude syndrome, is a rare disorder characterized by aberrant adipose tissue accumulations [1,2,3]. This adipose tissue hyperplasia is usually vast and symmetric, distributed usually along the facial, cervical, and abdominal regions, but cases have been reported involving inguinal and distal regions [1,2,3]. Epidemiologic studies report a higher prevalence in males rather than females, with a ratio of 15:1 to 30:1. Its onset is from the age of 20 onwards, and due to its low incidence rate (1 in 25,000 subjects), etiology as well as pathophysiology are yet to be elucidated [4]. Currently, MD diagnosis is reached through anamnesis, clinical examination, X-ray computed tomography, or magnetic resonance imaging. Moreover, though other studies report a lack in comorbidities, the disease has been frequently associated with alcoholism, liver disorders, diabetes, and dyslipidemia [5]. The only available treatment for this disease is surgery, through fat tissue excision, liposuction, or a combination of both but is sadly not definitive due to its high recurrence rate. To overcome this problem, new therapeutic strategies targeting pathogenetic mechanisms are requested. Indeed, several studies tried to elucidate the biology underneath the pathology without reaching a definitive conclusion [5,6]. In fact, it has been reported that MD tissues and cells show aberrant growth, mitochondrial energy metabolism, brown fat tissue alterations, and adipogenic index, ascribing these phenomena to specific proteins, such as Uncoupling Protein (UCP)-1 (a brown adipocyte marker) [7,8], calcyphosine-like (CAPSL) protein [9], or microRNAs such as miR-125a-3p and miR-483-5p, all associated with adipogenesis or fatty acid oxidation in peroxisomes [10].

Further research correlates MD with the tissue damages observed due to the high alcohol intake, suggesting that an enhanced inflammatory response could play a role in the onset of this disease [11]. Moreover, liver involvement and its link to the collapse of the lymphatic system have been correlated to the singular fat tissue accumulation location, such as the neck and thorax, sites full of lymphatic ducts [12]. For this reason, alcohol and other mediators of lymphatic vessel leakage may play an important role in MD. Finally, several studies linked the rise of this pathology with mitochondrial disfunction, and scientists have recently performed wide genome analysis on MD mitochondrial and genomic DNA, reporting an association of this disorder with myoclonus epilepsy and ragged red fibers (MERRF) mitochondrial tRNA(Lys) A > G (8344) mutation [13,14]. Others report mutations in the LIPE gene and MFN2 gene, associating MD with Charcot Marie tooth disease and lipodystrophy [15,16]. These associations are still dubious though due to their very low frequency in the population.

We thought that developing an in vitro model of the disease could help in getting further insights into the biological and molecular pathophysiology of MD, possibly contributing to novel strategies for disease diagnosis and therapy. In this regard, our group demonstrated in several neoplastic and nonneoplastic diseases the usefulness of isolating stem cells from diseased tissues to obtain an in vitro model recapitulating the pathological condition of the tissue of origin [17,18,19,20,21,22]. Specifically, we demonstrated that it was possible to isolate from human adipose tissue a population of adipose-derived stem cells (ASC) that not only could be a valuable source for regenerative medicine [23,24,25] but also, when isolated from patients affected by human Nieman Pick C disease, could represent an in vitro model resembling the features of diseased cells [17]. A similar approach has been recently utilized to create an in vitro model of lipedema [26,27].

For this reason, the aim of this study was to isolate ASC from Madelung patients (MD-ASC) and to characterize them in terms of surface phenotype, proliferation, and differentiation potential, using, as a reference, ASC obtained from healthy tissues. Subsequently, Madelung tissues and their respective MD-ASC cells were assessed by Sanger sequencing analysis for the presence of mitochondrial mutations. Finally, we assayed the effects of the MD-ASC secretome on proliferation and the immunophenotype of normal ASC.

## 2. Materials and Methods

Samples were collected by the Plastic Department of the Azienda Ospedaliero Universitaria of Udine after informed consent was obtained, in accordance with the Declaration of Helsinki, and with approval by the Independent Ethics Committee of the University of Udine (Parere 103/2011).

### 2.1. Tissue Donors

We performed a retrospective analysis to identify subjects who underwent liposuction for MD between 2014 and 2019 at the Plastic Surgery Department of the University Hospital of Udine. We examined medical history, clinical presentation, and comorbidities of *n* = 8 MD patients. Formalin-fixed, paraffin-embedded (FFPE) tissues suitable for histological and molecular evaluations were retrieved (*n* = 8).

### 2.2. Primary Cell Line Isolation, Culture, and Conditioning

Human adipose-derived stem cells (ASC) were isolated from lipoaspirates obtained from *n* = 3 MD patients (MD-ASC) and *n* = 3 healthy subjects (ASC) who underwent cosmetic procedures and were cultured as in [28]. MD-ASC were isolated from 3 out of the 8 patients previously analyzed for molecular and clinical features. These 3 MD patients were males, with a mean age of onset of 61 ± 7 years and showed cervical (2/3) and upper chest (1/3) fat accumulation. Briefly, lipoaspirates were enzymatically dissociated in a 0.025% collagenase type II solution (Worthington, Columbus, OH, USA) in Joklik modified eagle’s medium (Sigma-Aldrich, St. Louis, MO, USA) for 30 min at 37 °C. Collagenase activity was blocked by adding a 10% fetal bovine serum (GE Healthcare, Chicago, IL, USA) solution in Joklik modified eagle’s medium (Sigma-Aldrich, St. Louis, MO, USA). Cell suspension was centrifuged at 600× *g* for 10 min and filtered through a sieve (BD Falcon, Franklin Lakes, NJ, USA) in order to select a population less than 70 μm in diameter.

Freshly isolated human cells (2.0 × 10^6^) were plated onto 100-mm human fibronectin (Sigma-Aldrich, St. Louis, MO, USA)-coated dishes (BD Falcon, Franklin Lakes, NJ, USA) in an expansion medium composed as follows: 60% low glucose DMEM (Invitrogen, Carlsbad, CA, USA), 40% MCDB-201, 1 mg/mL linoleic acid-bovine serum albumin (BSA) 10**^−^**^9^ M dexamethasone, 10**^−^**^4^ M ascorbic acid-2 phosphate, 1× insulin-transferrin-sodium selenite (all from Sigma-Aldrich, St. Louis, MO, USA), 2% fetal bovine serum (StemCell Technologies, Cambridge, UK), 10 ng/mL human PDGF-BB, and 10 ng/mL human EGF (both from Peprotech EC, London, UK). The medium was replaced with a fresh one every 4 days. Once cells reached 70–80% of confluence, they were detached with TrypLE Express (Invitrogen, Carlsbad, CA, USA) and re-plated at a density of 1.0–2.0 × 10^3^ cell/cm^2^. Cells were cultured in expansion medium for at least three passages.

For conditioning experiments, conditioned media, representative of the MD-ASC secretome, were obtained by diluting 1:1 the supernatants of MD-ASC with fresh expansion medium. Specifically, when at 80% of confluence, MD-ASCs were added with fresh medium that was successively collected after three days of culture. Before dilution with fresh medium, MD-derived culture supernatants were centrifuged (3000× *g*, 20 min, 4 °C) in order to eliminate cells and cell debris and were filtered through 200 nM filters. In conditioning experiments, 3 groups of cells were compared: ASC, ASC conditioned by MD-derived conditioned medium, and MD-ASC.

### 2.3. Cell Proliferation Assays

To assess cell proliferation, MD-ASC and ASC at the third passage in culture were plated at a density of 3.0 × 10^3^ cells/cm^2^ in a 96-well plate. The medium was replaced with a fresh one every 4 days. Every 24 h for 7 days cells were stained with 1 µg/mL Hoechst 33,342, incubated for 15 min at 37 °C, and images were acquired using an epifluorescence Leica DMI 6000B microscope connected to a Leica DFC350FX camera (Leica Microsystems, Wetzlar, Germany). Cells at each time point were counted using ImageJ 1.52i software. Population doubling time (PDT) was calculated during exponential growth (log phase) with the following formula: PDT = T ln2/ln(Xe/Xb), where T is the time interval considered, Xb is the cell number at the beginning of the time interval, and Xe is the cell number at the end of the time interval. All experiments were performed in triplicate.

### 2.4. Flow-Cytometry

To assess MD-ASC and ASC surface immunophenotype, cells at the third passage in culture were detached using TRYPLE Express (Invitrogen, Carlsbad, CA, USA) and were incubated with properly conjugated primary antibodies: CD29, CD49a, CD49b, CD49d, CD9, CD144, CD44, CD117, CD59, CD38, HLA-ABC, KDR, and HLA-DR (BD Biosciences, San Josè, CA, USA); CD73, CD90, and CD34 (eBioscience, Paris, France); CD271 and CD146 (BD Pharmigen, San Josè, CA, USA); CD66e (Serotech, Milan, Italy); CD133 (Miltenyi Biotec, Bergisch Gladbach, Germany); ABCG-2 and CXCR4 (R&D, Minneapolis, MN, USA); CD51 and CD49f (BioLegend, San Diego, CA, USA); CD10 (AbD SEROTEC, Milan, Italy); and CD45 (Invitrogen, Carlsbad, CA, USA). Isotypic controls were assessed as negative. At least 2.0 × 10^4^ events were collected using FACS CANTO II (BD-Biosciences, San Josè, CA, USA) flow cytometer, analyzed using FACS Diva (BD-Biosciences, San Josè, CA, USA) software, and presented using FlowJo v10 (Becton Dickinson, San Josè, CA, USA) software.

### 2.5. Adipogenic Differentiation

MD-ASC (*n* = 3) and ASC (*n* = 3) were assessed for their differentiation potential toward adipogenic lineage. ASCs were differentiated and analyzed as described previously [23,28]. Specifically, adipogenic differentiation was induced by growing cells for 2 weeks at high density (2.0 × 10^4^/cm^2^) onto fibronectin-coated coverslips in 24-well plates in expansion medium added with 10% fetal bovine serum (GE Healthcare, Chicago, IL, USA), 0.5 mM isobutylmethylxanthine, 50 μM indomethacin, and 0.5 μM dexamethasone (all from Sigma-Aldrich, St. Louis, MO, USA). The medium was changed every 4 days.

The accumulation of lipid droplets indicating adipogenic differentiation was detected for staining cells in Oil Red-O stain solution as described by the manufacturers (Bio-Optica, Milan, Italy). Brightfield images were captured using Leica DMD108 microscope (Leica Microsystems, Wetzlar, Germany) at 20× magnification (numerical aperture: 0.70). Adobe Photoshop 20.0.3 software was utilized to compose and overlay the images and to adjust contrast (Adobe, San Josè, CA, USA). At least 200 cells were assessed. All experiments were performed in triplicate.

### 2.6. Immunofluorescence

ASC (*n* = 3) and MD-ASC (*n* = 3) cultured in differentiation medium were fixed in 4% buffered paraformaldehyde for 20 min at room temperature (R.T.). For intracellular staining, fixed cells were permeabilized for 10 min at R.T. with 0.3% Triton X-100 (Sigma-Aldrich, St Louis, MO, USA) before exposing them to primary antibodies. Unspecific binding was blocked using 5% BSA in phosphate-buffered saline (PBS) for 1 h at R.T., and cells were incubated overnight at 4 °C to detect Peroxisome Proliferator-Activated Receptor Gamma (PPAR-Gamma) (1:100; Santa Cruz Biotechnology. Inc., Heidelberg, Germany). A488 (Molecular Probe, Invitrogen, Carlsbad, CA, USA) was used as secondary antibodies and was diluted to 1:800 for 1 h at 37 °C. Finally, Vectashield (Vector-Labs, Burlingame, CA, USA) added with 0.1 μg/mL DAPI (Sigma, St Louis, MO, USA) was used as a mounting medium.

Epifluorescence images were acquired using a Leica DMI 6000B microscope (Leica Microsystems, Wetzlar, Germany) at 40× oil immersion (numerical aperture: 1.25). Adobe Photoshop software was utilized to compose and overlay the images and to adjust contrast (Adobe, San Josè, CA, USA). The differentiation potential was calculated by determining the fraction of ASC cells displaying either Oil Red-O or PPAR-Gamma positivity. At least 200 cells were assessed. All experiments were performed in triplicate.

### 2.7. DNA Extraction and Amplification of Mitochondrial Genome

Total DNA was extracted from either FFPE adipose tissue sections of MD patients (*n* = 8) or ASC at the third passage in culture (*n* = 3 ASC and *n* = 3 MD-ASC) using the QIAamp DNA Mini Kit (Qiagen, Hilden, Germany), according to the manufacturer’s recommendations. The extracted DNA concentration was determined by a spectrophotometer measurement (Nanodrop2000 Thermo Scientific™, Waltham, MA, USA) from the absorbance at 260 nm.

To separate mtDNA from linear DNA molecules, a mitochondrial specific amplification was carried out with the REPLI-g Mitochondrial DNA Kit (Qiagen, Hilden, Germany) loading 50 ng of total DNA template per 50 μL of final reaction buffer. The kit enables selective amplification of mitochondrial DNA from total DNA samples without the need for prior mitochondrial DNA isolation.

### 2.8. SNP Genotyping Analysis by Sanger Sequencing

The amplification reaction of mtDNA target fragments was performed by PCR using primers designed with the Primer blast software (NCBI-NIH). Specifically, the primers’ sequences were as follows:

mtDNA FW: 5′-TAGCATTAACCTTTTAAGTT
mtDNA RV: 5′-CCTTTAGTGTTGTGTATGGT

Each PCR reaction mixture was composed of 10 μL of 2× GoTaq^®^ Colorless Master Mix (Promega, Madison, WI, USA), 2 μL of mixed forward and reverse primers (10 µM each), 5 μL of template diluted 1:10, and 3 μL of nuclease free water. Thermal cycling conditions were as follows: 95 °C (5:00) + (94 °C (0:30) + 60 °C (0:30) + 72 °C (1:30)) × 40 cycles + 72 °C (10:00) + 4 °C (∞).

All PCR amplified products were loaded on a 2% agarose gel (*w*/*v*) to check the size of amplicons with reference to the 50 bp plus DNA ladder (Thermo Scientific™, Waltham, MA, USA); 5 μL of amplification products were cleaned up with the ExoSAP-IT Express (Thermo Scientific Waltham, MA, USA) reagent containing digestion enzymes to remove excess primers and dNTPs.

Sample preparation for sequencing was carried out with the Applied Biosystems™ (Foster City, CA, USA) BigDye™ Terminator v3.1 Cycle Sequencing Kit, in accordance with the manufacturer’s instructions. Briefly, each sequencing reaction contained 1 μL of BigDye Terminator ready reaction mix (Applied Biosystems Foster City, CA, USA), 10 pmoles of primer forward or reverse, 4 μL of 5× Sequencing Buffer, and 5 μL of PCR template for a total of 40 μL in volume. The cycle conditions were initial denaturation at 95 °C for 5 min followed by 35 cycles at 95 °C for 30 s, 55 °C for 20 s, and 60 °C for 4 min.

Excess of dye terminators were removed with DyeEx 2.0 Spin columns (Qiagen, Hilden, Germany); 2 μL of each sample was resuspended in 8 μL of formamide solution and denatured by heat. The entire volume was loaded on the 3500XL Genetic Analyzer (Applied Biosystems, Foster City, CA, USA) automated DNA sequencing instrument.

Analysis of mitochondrial DNA and single-nucleotide polymorphism (SNP) evaluation were performed using the FinchTV 1.4 software. The NC_012920.1 sequence of human mitochondrial genome was used as a reference [29].

### 2.9. Statistical Analysis

All data obtained have been described using the mean ± standard deviation and were tested for normal distribution using the Kolmogorov–Smirnov test.

Paired *t* test or Mann–Whitney test, as appropriate, were used to compare two groups, while for a linear trend, repeated-measurements one-way analysis of variance followed by the Bonferroni posttest or Kruskal–Wallis test followed by Dunn’s test were used, as appropriate, to compare more than two groups. *P* < 0.05 was considered significant (Prism, version 8.0.1; GraphPad Software, Inc., La Jolla, CA USA).

## 3. Results

### 3.1. Clinical and Histological Features of Madelung Patients

**Figure 1:**
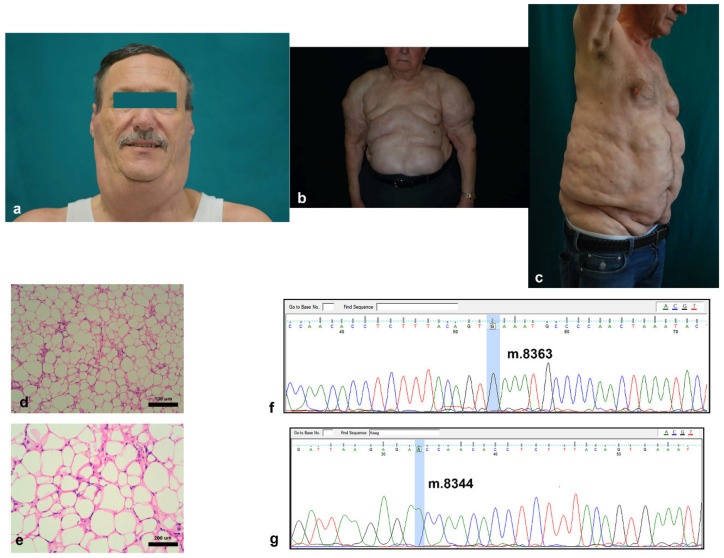
Clinical and histological features of MD: (**a**–**c**) representative pictures of typical anatomical localization of excessive adipose tissue in the cervical (**a**), upper trunk (the pseudoathletic appearance) (**b**), and abdominal (**c**) areas of 3 patients with MD; (**d**–**g**) representative pictures of hematoxylin and eosin stained adipose tissue sections of an MD patient ((**d**,**e**) at 20× and 40× magnification, respectively); and (**f**,**g**) representative Sanger pherograms of an MD-derived mitochondrial DNA lacking the presence of both m.8363G > A (**f**) and m.8344A > G (**g**) mutations (the interested DNA base positions are highlighted in light blue).

Adipose tissue sections obtained from MD patients (*n* = 8) were analyzed. Table 1 reports the demographics of the group. The majority of MD patients were males with upper trunk localization of fat accumulation, the so-called pseudo athletic appearance [30,31] (Figure 1a–c).

Madelung adipose tissues were characterized by smaller adipocytes with atypical nuclei and by thicker and more regular septa between adipocytes (Figure 1d,e).

Since several studies described the presence, in MD tissues, of some mitochondrial mutations, we tested the DNA isolated from 8 MD tissues for the presence of m.8363G > A and m.8344A > G, the two most frequently described variants. All MD samples showed a wildtype status for the mutations analyzed (representative electropherograms in Figure 1f,g).

### 3.2. Morphological and Functional Characterization of MD-ASC and ASC

**Figure 2:**
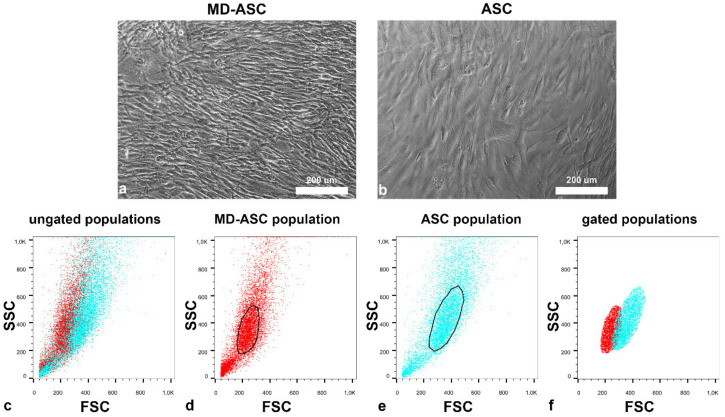
Morphology of MD-adipose-derived stem cells (ASC) and ASC cells: (**a**,**b**) phase contrast images of (**a**) MD-ASC and (**b**) ASC cell cultures, and (**c**–**f**) physical parameters (FSC and SSC) of both MD-ASC and ASC. The overlap in the scatter plots of the FSC and SSC of both MD-ASC (red dots) and ASC cells (blue dots) shows that the first ones are characterized by reduced FSC (**c**). Considering the (**d**) MD-ASC and (**e**) ASC scatter plots separately and drawing a gate around the core population, it was possible to better appreciate, when overlapping the gated populations, the differences in FSC and SSC of (**f**) MD-ASC and ASC.

ASC cells were efficiently isolated from lipoaspirates of *n* = 3 MD patients and compared to *n* = 3 healthy adipose tissue aspirates. As shown in Figure 2, data obtained from either microscopy (Figure 2a,b) or flow cytometry (Figure 2c,f) showed that MD-ASC cells (Figure 2d) were smaller than the non-MD ones (Figure 2e). Additionally, flow-cytometry showed that MD-ASCs were not only smaller (reduced forward scatter - FSC) but also less complex (reduced side scatter - SSC).

**Figure 3:**
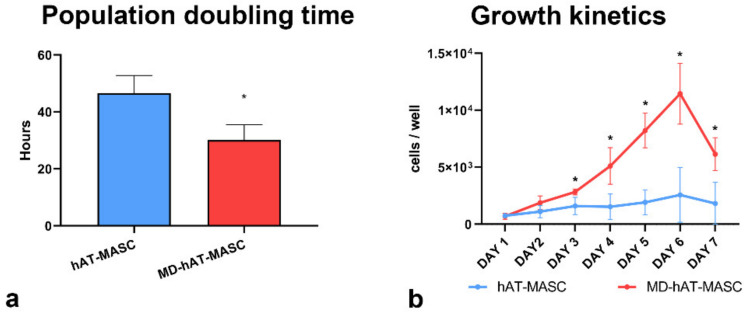
Proliferation of ASC cells: population doubling time (**a**) and growth kinetics (**b**). The results are expressed as mean ± standard deviation. * *p* < 0.05 vs. ASC.

The growth kinetics of MD-ASC and ASC were then evaluated by growth curve analysis. As reported in Figure 3a, the population doubling time (PDT) of MD-ASC was significantly shorter than that of ASC (30.1 ± 5.3 h versus 46.5 ± 6.2 h, respectively). Additionally, while ASC reached a plateau after 3 days, MD-ASC continued to grow with an exponential kinetic up to day 6 (Figure 3b).

These results suggest that MD-ASC cells have a significant increased growth potential and reached a steady phase at a higher cell concentration.

Given the differences in morphology and growth properties, MD-ASC and ASC were compared in terms of surface phenotype by analyzing a panel of twenty-six markers. As shown in Table 2, both cell populations display high expression levels of mesenchymal stem cell markers such as CD44, CD73, and CD90 and low expression of hematopoietic markers such as CD45 and CD38. Significant differences were detected in the expression of five markers. Specifically, MD-ASCs were characterized by reduced levels of stem cell markers CD90, CD9, and CD73. Regarding integrins, MD-ASC, with respect to ASC cells, presented increased levels of CD49a and reduced levels of CD49f (Table 2). Nearly significant but still noteworthy is the increased expression in MD-ASC of the pericyte marker CD146 [32].

Altogether, these results suggest that MD-ASC cells display a different surface phenotype, indicating an inconstant behavior, that possibly could explain the dissemination of fatty deposits that characterize the pathology.

**Figure 4:**
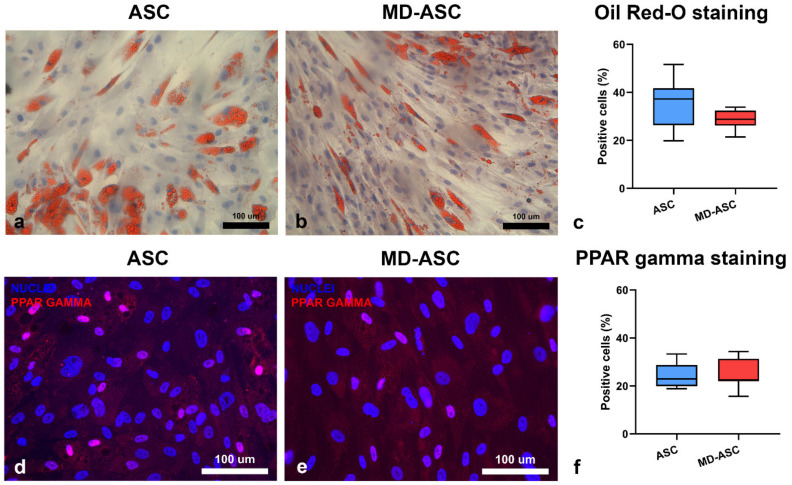
Adipogenic differentiation: (**a**,**d**) ASC and (**b**,**e**) MD-ASC were evaluated for their ability to differentiate into adipogenic lineage. Oil Red-O (red vacuoles, (**a**,**b**)) and peroxisome proliferator-activated receptor gamma (red, (**d**,**e**)) showed positivity. Nuclei are depicted by the blue staining of either hematoxylin or 4′,6-diamidino-2-phenylindole. (**c**,**f**) Histograms represent the fraction of cells positive for Oil Red-O staining (**c**) and expressing PPAR-Gamma (**f**). Data are expressed as mean ± standard deviation.

To understand if MD-ASC and ASC differed in their adipogenic potential, cells were exposed to specific differentiation-inducing conditions. Oil Red-O as well as PPAR-Gamma staining were performed to quantify the fraction of differentiated cells. As shown in Figure 4, no significant differences were detected between the two groups. Specifically, MD-ASC showed 28.72 ± 4% of Oil Red-O positivity while ASC cells showed 35.95 ± 10.2% Oil Red-O positivity. Similarly, the percentage of PPAR-Gamma-positive cells was 24.37 ± 6.3% in MD-ASC and 24.44 ± 5% in ASC. These data suggest that Madelung disease seems not to alter the in vitro differentiation potential of ASC.

Finally, MD-ASC as well as ASC cells were assayed for the presence of m.8363G > A and m.8344A > G mutations in the MTTK gene. MD-ASC, similarly to ASC cells, did not show tested mutations (Table 3).

### 3.3. MD-ASC Secretome Is Able to Trigger ASC/MD-ASC Switch

We have previously shown that MASC isolated from diseased tissues, either neoplastic [18,19] or nonneoplastic [20,21], are able to modify the phenotype of target cells through their secretome. Therefore, we wondered if MD-ASC cells were able to change the phenotype of normal cells through their secretome. For this purpose, ASCs (*n* = 3) were conditioned with the secretome of MD-ASCs (*n* = 3) in order to detect a change in their growth potential as well as in their phenotype.

**Figure 5:**
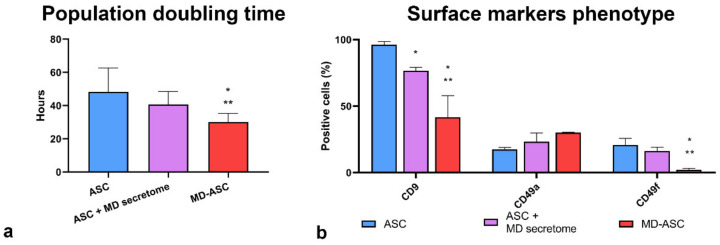
Influence of the MD-ASC secretome on ASC phenotype: (**a**) histogram comparing the mean population doubling times of ASC, ASC conditioned with MD-ASC, and MD-ASC and (**b**) surface marker expression comparison of CD9, CD49a, and CD49f in either ASC, ASC conditioned with MD-ASC secretome, or MD-ASC. Results are expressed as mean ± standard deviation. * *p* < 0.05 vs. ASC cells; ** *p* < 0.05 vs. ASC + MD-ASC secretome.

We first compared the population doubling time (PDT) of ASC with that of ASC conditioned by the supernatant of MD-ASC and of MD-ASC (Figure 5a). Although significant differences in the PDT were detected between MD-ASC and the other two conditions, we assessed a highly significant trend in the progressive PDT reduction from ASC to conditioned ASC and further to MD-ASC. In fact, ASC showed a PDT of 48.22 ± 14.49 h, while conditioned ASC and MD-ASC presented mean population doubling times of 40.7 ± 7.8 h and 30.1 ± 5.3 h, respectively.

We then evaluated by flow-cytometry the changes in the surface phenotype of ASC upon exposure for 72 h to the secretome of MD-ASC (Figure 5b). We took into consideration CD9, CD49f, and CD49a, since the first two were significantly downregulated in MD-ASC, with respect to ASC, and the third one was upregulated.

As shown in Figure 5b, the expression of the adhesion and migration marker CD9 was significantly reduced in conditioned ASC with respect to nonconditioned cells. Moreover, the CD9 marker showed a significant trend from the non-Madelung phenotype to the Madelung one. CD49f stem cell marker, though nonsignificantly different from conditioned and nonconditioned ASC, showed a statistically significant trend in reduction from ASC to conditioned ASC and further to MD-ASC. CD49a did not show a significant difference due to the high variability of the conditioned phenotype.

Altogether, these data suggest that the MD-ASC secretome could enable a switch in growth potential and surface phenotype of ASC cells, though this effect needs to be further investigated.

## 4. Discussion

MD is a rare disease characterized by the symmetrical accumulation of abnormal tumor-like subcutaneous adipose tissue. Due to the lack of unequivocal features or biomarkers, it remains an underdiagnosed and undertreated pathology [33]. Indeed, most of the research literature on the topic is represented by case reports. The pathogenesis of this disease has not been fully elucidated although it is believed that the MD phenotype may require a combined effect of alcohol (or other insults) and currently unknown genetic mutations [33]. In fact, the association of peculiar features of this disorder with different, often genetic pathologies seems to suggest the involvement of a genetic component, and in some patients, mutations of mitochondrial DNA have been reported [14].

In this work, we have analyzed clinical, histological, and molecular features of FFPE tissues retrieved from *n* = 8 Madelung patients surgically treated at the Plastic Surgery Department of University Hospital of Udine. Then, for the first time, to our knowledge, we isolated human adipose-derived stem cells from adipose tissue of 3 Madelung patients (MD-ASC) and compared them with *n* = 3 ASC obtained from healthy individuals in terms of growth kinetic, surface immunophenotype, and in vitro adipogenic differentiation. Finally, we evaluated the effect of the secretome of MD-ASC on healthy ASC cells to assess whether it possesses the ability to switch normal cells towards a MD phenotype.

In this study, we analyzed 8 patients with a clinical diagnosis of Madelung Disease who underwent liposuction to remove adipose tissue masses causing symptoms by compressing tissue structures and vessels. Patients presented abnormal tissue growth in several body parts, comprising distal regions, and thoracic and cervical areas among all. From a histological point of view, the adipose tissue of all patients was characterized by smaller adipocytes and by the presence of fibrotic and vascular enhancement but without atypia characteristic of lipomas [3]. Moreover, tissues were assessed for the presence of m.8363G > A and m.8344A > G, two mitochondrial DNA mutations reported as present in some MD patients [34,35]. None of the patients carried either of the two mutations taken in consideration. This data is still coherent with the reported 16% prevalence of the mutation in the Madelung population [36]. Still, it would be interesting to test a greater number of subjects in order to either confirm the prevalence reported in this work or to eventually find novel putative mutations related to the pathology.

Since we were previously able to develop in vitro models of neoplastic and nonneoplastic diseases [17,18,19,20,21,22], we isolated 3 ASC from lipoaspirates or surgical excisions of MD patients. Compared to healthy ASC, MD-ASC cells were smaller and grew faster and, in their growth kinetic, reached the steady state at a significant higher cell density. MD-ASC cells have subsequently been assessed for their immunophenotype. Both MD-ASC and ASC were characterized by an adipose-derived stem cell phenotype, being highly positive for CD73, CD44, and CD90 and negative for CD34, CD45, and HLA-DR. However, some differences could be detected: MD-ASC, with respect to their healthy counterpart, expressed less CD90, CD9, and CD49f and more CD49a. It has been hypothesized that CD90 could control the adipogenic differentiation of mesenchymal stem cells, since CD90 reduction favors differentiation [37]. In our case, despite a significant difference in CD90 expression, we did not notice a difference in the adipogenic differentiation. However, it is noteworthy that, in MD-ASC, the positivity for CD90 is high anyway and this can explain why differences in adipogenic differentiation were not detectable. CD73 is another mesenchymal stem cell marker described in both healthy ASC and in adipose-derived stem cells isolated from lipomas that is involved in differentiation, proliferation, and ATP metabolism [38]. The tetraspanin CD9 is a molecule involved in cell motility, adhesion, and differentiation. Kim et al. demonstrated a direct link between high CD9 levels and enhanced proliferation, adhesiveness, and in vivo engraftment [39]. The significant lower levels of CD9 reported in MD-ASC has never been reported in the literature as a feature of lipomas. Although considered a stem cell marker, it has been recently shown that downregulation of CD9 expression can protect from cellular senescence [40]. CD9 was shown to act through a phosphatidylinositide 3 kinase-AKT-mTOR-p53 signal pathway and it would be interesting in the future to investigate this pathway in MD-ASC. CD9 can associate with integrins, such as alpha-1 integrin (CD49a) and alpha-6 integrin (CD49f), which are also differentially expressed in MD-ASC when compared to ASC. Specifically, CD49f was downregulated and CD49a was upregulated. In the bone marrow, CD49a and CD49f have been reported to be discriminant for cells endowed with higher multipotency and stemness status [41]. However, it has been also shown that, in mammary stem cells, aging is associated with an increased expression of CD49f paralleled by a decline in function and increased transformation potential [42]. Regarding CD49a, it has been reported that selection using this integrin enhances the multipotentiality of mesenchymal stem cells [43]. We have instead noticed and increased the expression of CD49a in mesenchymal stem cells isolated from diseased tissues. Considering the trend toward an increased expression of CD146 in MD-ASC, it has been shown that, in mesenchymal stem cells from the umbilical cord, an increased expression of CD146 is associated to higher angiogenic and vasculogenic potential [44]. It would be interesting to explore the link with increased vascularization of fat deposits in MD patients.

Our studies so far characterized the cells based on their surface phenotype, but further investigations could include wide genome and proteomic analysis.

One of the main features of adipose-derived stem cells is the ability to differentiate once exposed to specific differentiation-inducing conditions [45]. To better understand putative differences between MD-ASC and ASC, we induced differentiation into adipogenic lineage. The data showed no significant differences in adipogenic differentiation, assessed by Oil Red-O and PPAR-Gamma staining, between MD-ASC and ASC. Considering the adipogenic differentiation capacity of ASC isolated from other diseased tissues, there are variable results in the literature. For example, considering lipoma-derived stem cells, with respect to healthy adipose tissue-derived stem cells, Stojanovic et al. reported a decreased level of adipogenic differentiation [46] while Teshima reported, in dogs, an opposite trend [47]. Moreover, Al-Ghadban et al. demonstrated an enhanced adipogenic differentiation potential of lipedema ASC compared to healthy ASC [26].

Our results, focused for the first time on MD-ASC, showed no major differences between MD-ASC and ASC. However, the evaluation of a single differentiation lineage constitutes a limitation of this study, and it could be interesting to evaluate the differentiation potential towards other cell lineages, such as chondrogenic and osteogenic lineages, to better clarify the full differentiation potential of the MD-ASC.

As Madelung disease is referred to as a systemic disorder, we wondered if there could be a connection with the disseminated features of the pathology and the secretome of these cells. We have demonstrated in several disease settings that stem cells isolated from diseased tissues can influence, through their secretome, the phenotype and function of healthy mesenchymal stem cells, favoring a switch toward an aberrant phenotype [18,19,48]. For this reason, we conditioned healthy ASC with the supernatant of MD-ASC and assessed proliferation and surface markers phenotype. After 3 days of treatment, conditioned cells showed a significant trend toward a reduction in the PDT. Regarding the phenotype, conditioned cells were characterized by a significant reduction in CD9 and a trend towards the reduction of CD49a and the increase of CD49f. This suggests that the secretome of MD-ASC can induce in normal cells an aberrant phenotype, thus extending the disease beyond the borders of diseased cells.

The secretome of cultured cells is constituted of different constituents, such as cytokines, released proteins, growth factors, extracellular vesicles, and nucleic acids. The effect of the ASC secretome on cells and tissues has been widely studied for both regenerative medicine and immune-modulating approaches, as it is a cell-free approach and, for this reason, is safer and less restricted with respect to cell-based therapies. However, conflicting results have been described, partly due to different cell sources, culture, and experimental settings. An important next step would be to identify which component of the Madelung secretome is responsible for the “activated” phenotype seen in Madelung patients as it could be a putative candidate for target therapy. For instance, the modulation of CD9, a key exosome tetraspanin, might suggest a role of extracellular vesicles and, specifically, of exosomes. However, it could be interesting to extend analyses also to other soluble factors, such as cytokines or chemokines, or circulating nucleic acids. Wide spectrum analysis of the constituents of both MD-ASC and ASC can contribute to identifying factors with roles responsible for the insurgence and recurrence of the pathology that must be in any case demonstrated through proper functional assays.

In conclusion, in this paper we have shown that ASC can be isolated from Madelung adipose tissues and that they show, with respect to their healthy counterpart, some phenotypic and functional differences. Importantly, the secretome of MD-ASC can favor a switch towards an aberrant phenotype of ASC, suggesting a possible mechanism of propagation of the pathology from diseased tissues to healthy tissues. Taking advantage of this patient-based in vitro model can help in dissecting the mechanisms underlying this so far uncurable pathology, possibly suggesting novel therapeutic strategies.

## Figures and Tables

**Table 1:** Madelung disease (MD) patient demographics.

		Madelung Patients
*N*. of subjects		8
Age of onset (years) (mean ± SD)		49 ± 25
Sex; *n* (%)		
	Male	6 (75%)
	Female	2 (25%)
BMI (body mass index) (kg/m^2^) (mean ± SD)		29.8 ± 3.7
Localization of excess fat; *n* (%)		
	Cervical	2 (25%)
	Upper chest	4 (50%)
	Abdominal	2 (25%)
Smoker; *n* (%)		
	Yes	2 (25%)
	No	6 (75%)
Comorbidities; *n* (%)		
	Hepatic disease	3 (37.5%)
	Hypertension	2 (25%)
	Diabetes mellitus	2 (25%)
	Overweight	4 (50%)
	Hyperuricemia	3 (37.5%)
	Hyperthyroidism	1 (12.5%)
	Obstructive sleep apnea	2 (25%)
	Peptic ulcer	4 (50%)

**Table 2:** Cell surface markers of MD-ASC and ASC.

	MD- ASC	ASC	*p*-Value
CD90	94.43 ± 2.15	99.9 ± 0.01	0.011789
CD44	91.36 ± 8.80	99.93 ± 0.05	0.167086
CD29	92.56 ± 9.18	100 ± 0	0.233574
CD73	82.16 ± 4.19	98.73 ± 1.59	0.006143
CD49B	87.5 ± 9.89	99.4 ± 2.17	0.106926
CD51	89.6 ± 9.49	99.93 ± 0.05	0.132482
CD59	64.86 ± 30.80	90 ± 3.9	0.231021
CD146	45.23 ± 13.00	21.73 ± 11.35	0.077795
CD49D	46.75 ± 3.04	57 ± 5.65	0.174691
CD10	69.6 ± 14.71	51.56 ± 6.85	0.126587
CD9	32.25 ± 0.35	96.16 ± 2.4	0.004540
CD49A	30.05 ± 0.21	17.35 ± 1.48	0.006903
CD49F	2.13 ± 1.02	20.76 ± 4.98	0.003157
CD66E	6.26 ± 3.32	1.2 ± 0.70	0.136762
CD45	1.33 ± 0.72	2.16 ± 1.87	0.512728
CXCR4	4.56 ± 4.7	2.76 ± 3.93	0.637976
CD38	0	0	-
CD144	0	0	-
CD271	0	0	-
CD133	0	0	-
HLA-ABC	0	0	-
CD117	0	0	-
CD34	0	0	-
ABCG2	0	0	-
KDR	0	0	-
HLA-DR	0	0	-

**Table 3:** Mitochondrial DNA concentration and mutational status.

	Concentration	m.8363G ≥ A Status	m.8344A ≥ G Status
ASC 1	23 ng/µL	Wildtype	Wildtype
ASC 2	50 ng/µL	Wildtype	Wildtype
ASC 3	15 ng/µL	Wildtype	Wildtype
MD-ASC 1	41 ng/µL	Wildtype	Wildtype
MD-ASC 2	18 ng/µL	Wildtype	Wildtype
MD-ASC 3	34 ng/µL	Wildtype	Wildtype

## Data Availability

Data available on request from the corresponding author due to restrictions e.g., privacy or ethical.
